# "Any other comments?" Open questions on questionnaires – a bane or a bonus to research?

**DOI:** 10.1186/1471-2288-4-25

**Published:** 2004-11-08

**Authors:** Alicia O'Cathain, Kate J Thomas

**Affiliations:** 1 Medical Care Research Unit, Health Services Research, School of Health and Related Research, University of Sheffield, Regent Court, Sheffield, UK

## Abstract

**Background:**

The habitual "any other comments" general open question at the end of structured questionnaires has the potential to increase response rates, elaborate responses to closed questions, and allow respondents to identify new issues not captured in the closed questions. However, we believe that many researchers have collected such data and failed to analyse or present it.

**Discussion:**

General open questions at the end of structured questionnaires can present a problem because of their uncomfortable status of being strictly neither qualitative nor quantitative data, the consequent lack of clarity around how to analyse and report them, and the time and expertise needed to do so. We suggest that the value of these questions can be optimised if researchers start with a clear understanding of the type of data they wish to generate from such a question, and employ an appropriate strategy when designing the study. The intention can be to generate depth data or 'stories' from purposively defined groups of respondents for qualitative analysis, or to produce quantifiable data, representative of the population sampled, as a 'safety net' to identify issues which might complement the closed questions.

**Summary:**

We encourage researchers to consider developing a more strategic use of general open questions at the end of structured questionnaires. This may optimise the quality of the data and the analysis, reduce dilemmas regarding whether and how to analyse such data, and result in a more ethical approach to making best use of the data which respondents kindly provide.

## Background

The survey is a key method in health services research [1]. The majority of survey questionnaires consist of closed questions where respondents are asked to choose from a fixed number of options. These are considered to be efficient because data are easy to collect, code and analyse [2]. Efficiency is important in survey methodology where researchers attempt to obtain the attitudes or experiences of a representative sample for generalisation to a wider population, and may need to gather information from large numbers to ensure precision of estimates. In addition to closed questions, it is not uncommon to include an 'open' question where respondents are invited to provide information in free text format, for example 'Is there anything else you would like to say' at the end of a questionnaire. When the questionnaires are returned and being prepared for analysis, the researcher may face the dilemma of whether or not to analyse and report the written responses to this open question. In this paper we draw on expert opinion in key texts, and examples of the use of open questions in predominantly closed question questionnaires, to consider whether there is value in including such questions, and if so, how best to optimise the quality of the data and analysis.

## Discussion

### Different types of open questions in surveys

**Figure 1:**
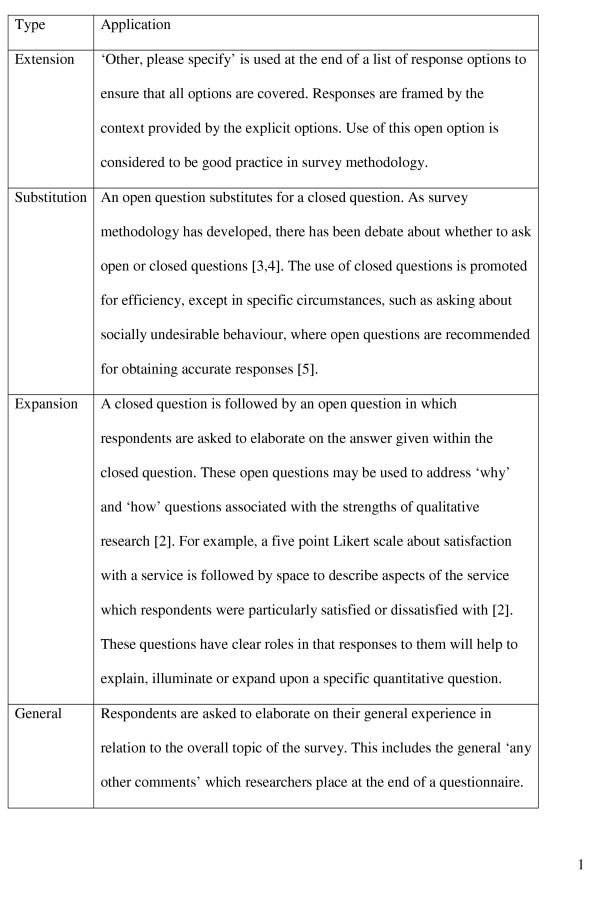
Different types of open questions in surveys

There are four types of questions which might require an open rather than closed response (see Figure 1). The general open question, typically 'any other comments?', used at the end of a structured questionnaire is the type we focus on in this paper. We believe that the use of this type of open question is common, and we consider it to be the type that is most likely to pose a dilemma for researchers around whether and how to analyse any responses.

### The potential benefits of general open questions

General open questions offer a number of benefits when piloting a questionnaire. Responses to them can reassure the researcher that all relevant issues have been covered [3-5]. Responses may also be used to corroborate answers to closed questions, offering reassurance to the researcher that the questionnaire is valid, or highlighting problems with particular questions.

The benefits of using general open questions in the main study are less clear. They have been recommended to help make a dull statistical report more interesting [6], by providing the reader with quotes to illustrate important points, and in self administered questionnaires because there is some evidence that they increase response rates [5]. Increasing the response rate is a considerable benefit in survey methodology, but it is not necessarily the issue which drives researchers to use general open questions.

Researchers may use general open questions without giving much thought to why they are doing so, simply including the question because it is usual practice. Or they may be driven by a desire to offer respondents an opportunity to voice their opinion. Closed questions represent the researchers' agenda, even if they have been developed through listening to people's views in focus groups and depth interviews. The use of 'any other comments' may redress the power balance between researchers and research participants. Respondents may take this opportunity to ask for clarification or information about a health issue or health service, or voice concern about the research. If researchers include a general open question for this reason then they will need to consider how best to respond to individuals about such queries and concerns.

Another possible driver for including a general open question is a concern about missing an important issue, even if the questionnaire has been developed using a considerable amount of qualitative research and piloting. There may be issues which respondents want to give more details about than the structured questions allow. There may be issues which qualitative methods and piloting fail to uncover because they affect a small number of people only, or they are specific to sub groups which have not been included in the development work, or they have occurred since the design of the questionnaire. Thus general open questions may act as a 'safety net' and help the researcher to identify issues not covered by the closed questions, either by elaborating and explaining some of the findings from closed questions, or identifying new issues.

For example, the purpose of a survey of NHS Direct nurses was to describe the nurses' qualifications, experience and reasons for joining the service [7]. A small number of closed questions asked about nurses' views of working for this new service, and respondents used the general open question to expand in considerable detail on this issue [8]. In other studies, responses to general open questions have elaborated on answers to a closed question, identifying the aspects of the service which contributed to NHS Direct users feeling reassured by the advice offered [9], explaining why junior doctors felt that training had not prepared them for their job [10], and illuminating why people were more satisfied with an emergency ambulance call taker making use of a priority dispatch system [11]. An example of a new issue emerging after the design of the questionnaire was the emergence of media criticism as a concern of doctors in one of a series of annual surveys [12].

### Why are general open questions a problem?

Having asked a general open question, researchers may face the dilemma of whether to analyse responses or not. Practical constraints may contribute to a decision not to do so because data input and analysis require considerable resources [5,6] and these may not have not been allocated during the study design. However, ignoring this data can feel unethical and it has been recommended that researchers should not ask open questions unless they are prepared to analyse the responses [13]. Another barrier may be the lack of clarity around the status of the responses. They tend to fall between two stools, being neither strictly qualitative nor quantitative data, and this can make them uncomfortable to work with. This lack of clarity of status may result in them not being analysed, or being analysed and published in the body or appendix of a report but not within any peer reviewed articles emerging from the study.

### Are responses to general open questions qualitative or quantitative data?

Some researchers consider responses to general open questions to be qualitative data [14,15], some do not [16], and others describe them as 'quasi-qualitative data' [17]. General open questions have some of the features of qualitative approaches: they appear to allow respondents to write whatever they want in their own words, with little structure imposed by the researcher; the output is words rather than numbers or ticks; the analysis may use techniques associated with qualitative research; and publication can involve the display of verbatim quotes so that it looks like qualitative data. However, data from general open questions can lack some of the key strengths of qualitative research. One could argue that the closed questions indicate the legitimate agenda for the responses to the general open question, and thus may impose constraints on responses. More importantly, there is a lack of attention to context, and a lack of conceptual richness, because the data on each case often consist of a few sentences or less. Typically, recipients are asked non-directive questions such as 'Is there anything else you would like to say' or 'Any other comments?', with a small amount of space for responses.

The key to determining the status of data derived from general open questions may therefore be their depth; both the amount recipients are prompted to write (either through the instructions given or the amount of space allocated), and the amount they actually write. Thus researchers may be able to determine the status of a general open question at the design stage of a study by having a strategy to generate depth and treat the data qualitatively, or by having a strategy to generate shorter responses as a 'safety net' for complementary or new issues. Having such a strategy may help researchers to devise a strategy for analysis and publication.

### Generating qualitative data by design

Researchers can determine the status of a general open question at the design stage of a study by having a strategy to generate depth and treat the data qualitatively. For example, at the end of a structured questionnaire about use of Chinese medicine, one researcher invited respondents to tell the 'story' of their use of Chinese medicine, leaving a full one and a half pages of white space, and offering a example of the detail required. The following instructions were given: 'Now tell us your own story, using the space on the next page. We've provided one true patient story to give you an idea of the kinds of details we need. The important subjects are repeated in the list above the space we've provided for you to write in. Also use the back of the page if you wish. Please remember to write clearly.' [18]. This approach produced 460 accounts from 575 respondents (80%). These 'handwritten stories' were treated as qualitative data, and analysis focused on the language used by respondents, as well as emerging themes, to show the holistic nature of the health care delivery as experienced by the respondents.

In the above example, the 80% response suggests that the stories obtained could be viewed as representative of the population surveyed. However, in qualitative research the validity of the study does not rest on the researcher's ability to demonstrate representativeness with respect to the total population. Rather, it rests on transferability whereby the researcher offers detailed description of the setting in which the research was undertaken [19]. Thus what is required is that the characteristics of the sample are clearly presented, such that the reader is informed about the likely transferability of the beliefs and experiences expressed. With data obtained from a structured survey, it  is always possible to use the quantitative responses to characterise the nature of the group providing comments, and to make their relationship to the wider population apparent. This means that comments from a subset of responders are still valuable data even when they do not represent the entire sample. One important corollary of this is that open questions can be designed expressly to elicit comments from a subset of the population surveyed, using the principles of purposive sampling [20]. An example of this would be to encourage all respondents reporting a particular type of experience in a closed question to tell their story. An alternative approach would be to sample post hoc from the full range of responses received, for example sampling information rich cases or extreme cases.

If the open question is used to generate qualitative data, then researchers will need to use qualitative analysis techniques and possibly qualitative software as used in the Chinese medicine example discussed previously [18], and will need to consider issues important to good quality qualitative research, such as clear exposition of data collection and analysis, the search for disconfirming evidence and reflexivity [17,21,22]. Qualitative researchers expect analysis to be challenging and time consuming and will ensure that they have the resources required to undertake it if the intention to collect such data is explicit in the research proposal. When reporting research findings from studies using face-to-face interviews, it is good practice to indicate the length of the interviews. Similarly, when reporting the data from these open questions, it might be helpful to indicate the potential depth of data to the reader by detailing the average number of lines of text available from respondents [18].

### Generating quantifiable data by design

General open questions may produce little more than the closed questions on the questionnaire [23] and rather than considering it unethical to analyse these responses, a more appropriate strategy might be preliminary analysis involving reading the responses so that the researcher can consider the contribution they make to the study overall. If the comments merely corroborate or slightly elaborate upon the answers to closed questions, then formal analysis may not be worthwhile [23]. It may be good practice to report within publications that the responses to the general open question did not provide additional information to the closed questions. It is where they offer insights or issues not available in the closed questions that formal analysis could be considered good practice, even if the role of this analysis is to identify hypotheses or questions for further study. Formal analysis may be prompted by either the strength of numbers making particular comments, or the strength of feeling within a small number of the comments. For example, in a survey of NHS Direct nurses, the large number of detailed comments and the emotional content of some of them, prompted a formal analysis [8], and in a survey of junior doctors, the strength of feeling expressed by a small number of doctors around one issue prompted formal analysis [12].

From a quantitative perspective, the strength of a survey approach is representativeness, and thus non-response bias should be a concern. Respondents are less likely to complete a general open question than a closed one on a postal questionnaire [4]: 81% of the 71% of respondents to a survey of NHS Direct users, that is 58% of the sample [9]; 67% of the 74% of respondents to a survey of NHS Direct nurses, that is 50% of the sample [8]; and 40% of the 74% of respondents to a survey of junior doctors, that is 30% overall [12].

Those who choose to answer the general open question could be different from respondents overall, either being more articulate or having a greater interest in the survey topic. It is important to consider and report on who has made written comments so that bias can be considered. In a patient satisfaction survey, females were more likely to make comments than males but interestingly there were no significant differences by age group or educational status [23]. In a survey of NHS Direct nurses, the proportion of nurses making written comments varied by their job satisfaction levels, with nurses who felt that their job satisfaction had 'not really changed' under-represented in the written comments and those who felt it had 'worsened a lot' over-represented in the written comments [8]. The comments were reported in this context.

Formal analysis must be rigorous so that the findings are useful and convincing. Content analysis may be undertaken [2,3], where the researcher takes the following steps:

1. Reads a sub-set of the comments.

2. Devises a coding frame to describe the thematic content of the comments.

3. Assigns the codes to all the comments. The coding frame can be applied using software designed for this purpose [24] or manually. Two coders may be needed to test the reliability of assigning codes [2].

4. The codes can be entered into a statistical package alongside the data from the closed questions and treated as variables in a quantitative analysis.

The coding process is time consuming and requires expertise [2,4,6]. The skills of a qualitative researcher are not needed, but the coding is similar to the early stages of qualitative analysis [25,26] and researchers may wish to seek the advice of a qualitative researcher. Any decisions made will affect the results and thus the coding process is suited to a skilled researcher.

When reporting the responses to general open questions it is important that the numbers of respondents making each comment are displayed, with recognition that although a specific number of people mentioned an issue it might be relevant to many more who did not choose to mention it. Although numbers rather than percentages tend to be used when reporting responses to open questions [8,12,15,26], percentages will sometimes be the most appropriate way of presenting the results, for example, in a before and after design with different numbers of comments in each time period [11]. Verbatim comments can be displayed to illustrate the themes [8,26], because it is the comments themselves which have convinced the researcher of the importance of the dissemination of the information [8]. When doing this, attention to confidentiality is important, taking care not to report comments which might identify an individual.

### Publication of responses to open questions

Publication of responses to open questions can occur within a paper reporting the main findings of the questionnaire or as a separate publication. Where comments elaborate and explain findings from closed questions, it may be most appropriate to publish them in the same paper [9]. Where a new issue emerges [8,12], a separate publication may be appropriate. For publication, both the data and analysis need to be robust enough to stand up to scrutiny and peer review.

### Advantages and disadvantages of having an explicit strategy

An explicit strategy requires that researchers consider the role of a general open question in the context of their survey, and its status in terms of generating either qualitative or quantifiable data. If the role of the question is to give a voice to participants then the researcher can ensure that comments will be read to identify any concerns and queries expressed by individuals, and that appropriate action is taken with individual comments. If the role is to generate qualitative data then attention can be paid to generating depth of data and the quality issues associated with qualitative research. If the role is to act as a safety net and generate quantifiable data then resources will need to be allocated for reading the comments, and if there appears to be added value, for formally analysing the data with attention to non-response bias and reliability of coding. Having a strategy may reduce any dilemma faced by researchers about whether and how to analyse these questions, may help the researcher to allocate the appropriate time and expertise to this data, and may produce an analysis robust enough for publication in peer reviewed journals. A potential disadvantage of having such a strategy may be that some flexibility is lost and that some important issues are missed. Finally, researchers cannot assume that they know how best to facilitate a respondent to complete a general open question and may need to consider using cognitive aspects of survey methodology to construct the question [27].

## Summary

• General open questions at the end of structured questionnaires can present a problem to researchers who may face the dilemma of whether or not to analyse them.

• They are necessary when piloting questionnaires because they identify further issues for inclusion in the survey, and may be a bonus in the main study because they may increase response rates and may identify issues which complement responses to closed questions.

• The value of such questions, and the quality of the data and analysis, may be optimised if researchers make more strategic use of them by being clear about their role, and understanding the type of data they wish to generate when they design their study.

• An explicit strategy for generating qualitative data will encourage attention to depth of data and issues important to the analysis of qualitative data such as reflexivity.

• An explicit strategy for generating quantifiable 'safety net' data, that is important issues missed by the closed questions, will encourage attention to non-response bias and reliability of coding.

## Competing interests

The author(s) declare that they have no competing interests.

## Authors' contributions

AOC conceived the paper and wrote the first draft. KJT reviewed it critically, and developed the sections on qualitative research. Both authors produced the final draft and read and approved the final manuscript.

## Pre-publication history

The pre-publication history for this paper can be accessed here:


